# Effect of Li^+^ doping on the luminescence performance of a novel KAlSiO_4_:Tb^3+^ green-emitting phosphor

**DOI:** 10.1038/s41598-021-84220-x

**Published:** 2021-03-04

**Authors:** Reziwanguli Yantake, Muyasier Kaiheriman, Taximaiti Yusufu, Aierken Sidike

**Affiliations:** 1 College of Physics and Electronic Engineering, Xinjiang Normal University, Urumqi, 830054 Xinjiang China; 2 Key Laboratory of Mineral Luminescent Material and Microstructure of Xinjiang, Urumqi, 830054 Xinjiang China; 3 Key Laboratory of New Light Source and Micro-Nano Optics, Urumqi, 830054 Xinjiang China; 4 Urumqi Campus of Army Academy of Border and Coastal Defense, Urumqi, 830002 Xinjiang China

**Keywords:** Structural materials, Materials science

## Abstract

A new green-emitting phosphor, KAlSiO_4_:1.5 mol% Tb^3+^, *x* mol% Li^+^, was prepared via a high-temperature solid-phase method, and its crystal structure, diffuse reflectance spectrum, and luminescence were studied. The results show that the Li^+^ doping shifts the strongest diffraction peak to a high angle direction, reducing grain size by 11.4%. The entry of Li_2_CO_3_ improves the luminescence performance of KAlSiO_4_:1.5 mol% Tb^3+^. At a Li^+^ concentration of 1.5 mol%, the sample has strong absorption in the ultraviolet light range from 250 to 400 nm. The luminous intensity of the sample at 550 nm approximately quadruples after Li^+^ doping. Additionally, the colour purity of the sample and the internal quantum yield increase to 83.3% and 42%, respectively. The sample changes colour with time when exposed to air without an obvious fading phenomenon. The emission intensity at 200 °C is 95.1% of its value at room temperature, indicating that the phosphor has excellent thermal stability when *x* = 1.5. These results show the feasibility of using the silicate phosphor for generating the green light component of white light-emitting diodes for solid-state lighting.

## Introduction

In recent years, rare earth luminescent materials have been widely used in white light-emitting diodes (w-LEDs) because of their long life and high efficiency^1^. In commercial solid-state lighting, phosphor-converted LEDs (pc-WLEDs) offer many advantages including low cost, good colour rendering, high colour purity, and good chemical stability. pc-WLEDs have also attracted the attention of many researchers^2^.

Silicate phosphor luminescent materials for LED lighting are made of cheap raw materials. They offer good thermal stability and have a long life. Muyasier et al. prepared a green Na_8_Al_6_Si_6_O_24_Cl_2_: *x* mol% Tb^3+^ phosphor and analysed the luminescence spectrum and colour coordinates, and they found that the silicate phosphor has a lifetime of up to 2.8 ms with good thermal stability^3^. Wan et al. prepared BaAl_2_Si_2_O_8_:Tb^3+^, Ce^3+^ phosphors, studied their photoluminescence characteristics, and found that they can be used as green phosphors^4^. Yu et al. prepared Sr_2_SiO_4_:Tb^3+^, Ce^3+^ phosphors with a lifetime of up to 2.85 ms. The colour of this phosphor can be changed from green to blue by using the high-temperature solid-phase method^5^. Dilare et al. prepared NaAlSi_3_O_8_:Tb^3+^ phosphor and obtained a new type of silicate luminescent material with tuneable colours from green to red^6^. KAlSiO_4_ has potential applications in luminescent glass and optical devices. In recent years, a few groups have reported on artificially synthesised KAlSiO_4_ doped with other rare earths to prepare three-color phosphors suitable for white LEDs.

Since Tb^3+^ has a strong green emission band in the wavelength range of 535 nm–555 nm, rare earth luminescent materials doped with Tb^3+^ can often be used to obtain the green component of an ideal white LED^7^. The actual luminous intensity of Tb^3+^ is generally determined by the crystal field coordination environment in the selected matrix. When different valencies are substituted, Li^+^ can often be used as a charge compensator or a flux to achieve the desired luminous efficiency. Li Zhen et al. reported a new green emitting phosphor Sr_2_MgB_2_O_6_:Tb^3+^, *x* mol% Li^+^ with good thermal stability and a maximum doping concentration of 9 mol%^8^. Potassium aluminosilicate (KAlSiO_4_) has a lattice site suitable for rare earth doping, and the corresponding crystal symmetry is relatively high. The standard card number of this material is 33–0989. In this study, a new type of green-emitting phosphor, KAlSiO_4_: 1.5 mol% Tb^3+^, *x* mol% Li^+^, was synthesised by a high-temperature solid-phase method. The crystal structure, photoluminescence characteristics, UV–Vis absorption spectrum, colour coordinates, and colour purity of the samples were systematically studied. The addition of Li^+^ improves the luminescence performance of KAlSiO_4_:1.5 mol% Tb^3+^, making it a new type of green silicate phosphor with potential application as the green component of white LEDs.

## Experimental results and discussion

### Phase analysis of KAlSiO_4_: 1.5 mol% Tb^3+^, ***x*** mol% Li^+^ series samples

**Figure 1:**
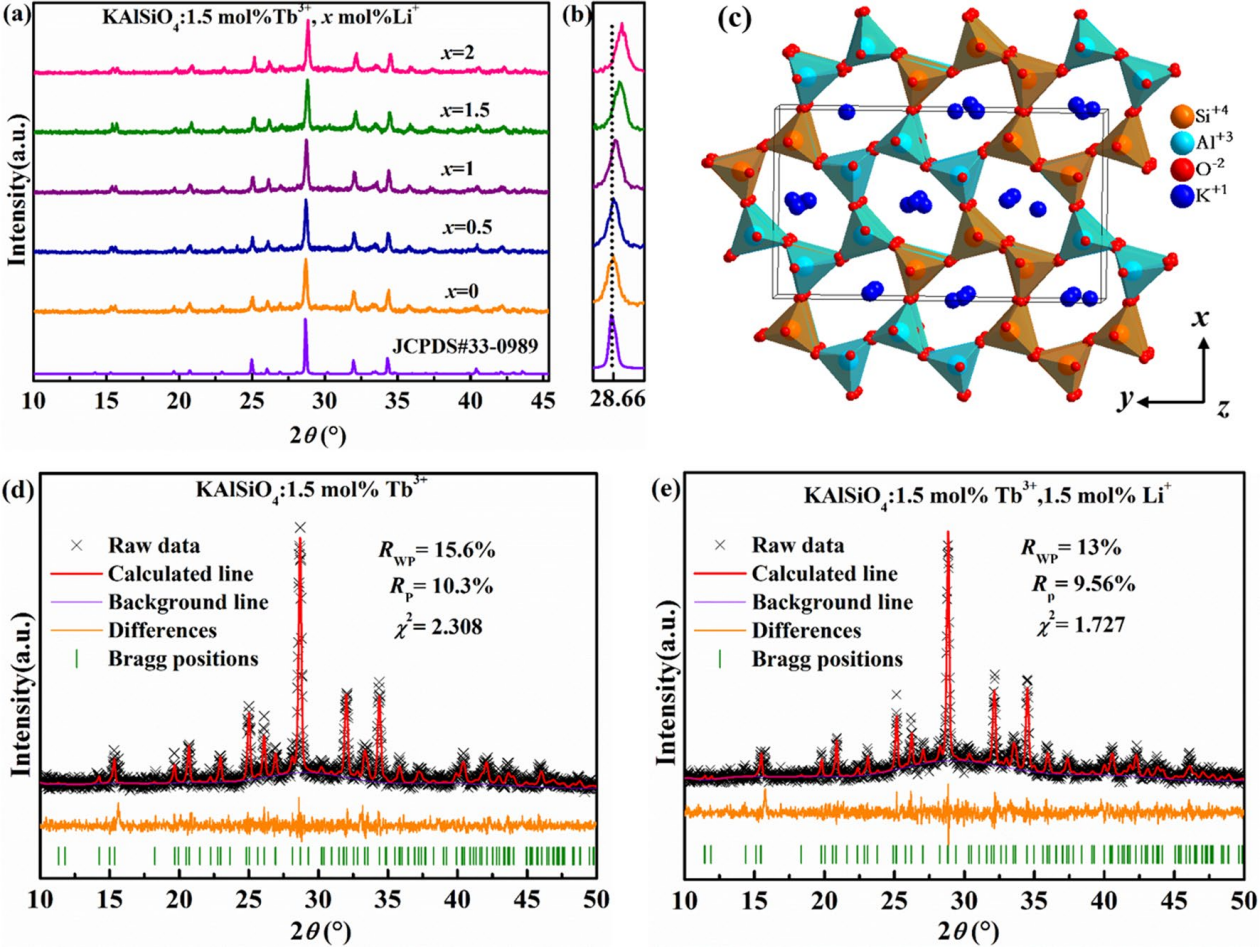
(**a**, **b**) X-ray diffraction pattern of KAlSiO_4_:1.5 mol% Tb^3+^, *x* mol% Li^+^. (**c**) Crystal structure of KAlSiO_4_. (**d**) Refined XRD image of KAlSiO_4_:1.5 mol% Tb^3+^ and (**e**) KAlSiO_4_:1.5 mol% Tb^3+^, 1.5 mol% Li^+^.

Figure 1a shows the X-ray diffraction pattern of the KAlSiO_4_:1.5 mol% Tb^3+^, *x* mol% Li^+^ (*x* = 0, 0.5, 1, 1.5, 2) sample. It can be seen from the figure that the diffraction peaks are consistent with the standard JCPDS No. 33–0989. Figure 1b shows an enlarged view of the strongest diffraction peak at 28.66°. The peak appears to gradually shift to a high-angle direction. Both Li^+^ and Tb^3+^ are added to the crystal lattice. It can be inferred that Li_2_CO_3_ can be used as a charge compensator in the KAlSiO_4_ crystal lattice to adjust the valence state of ions to achieve charge balance^1^. When CN = 7, Tb^3+^ (r = 0.098 nm) and Li^+^ (r = 0.092 nm) are close to the seven-coordinate K^+^ radius (r = 0.146 nm) in KAlSiO_4_, whereas Si^4+^(r = 0.026 nm) and Al^3+^ (r = 0.039 nm) have smaller radii. Hence, Tb^3+^ and Li^+^ replace the K^+^ positions. According to the Bragg formula [Eq. (1)]^6^, the interplanar spacing (d) will be reduced after the substitution. Hence, the diffraction peak position moves to a higher angle direction.

1 $$2d\sin \theta = n\lambda$$

where *θ* is the Bragg angle of X-ray diffraction peak, d is the interplanar spacing, n stands for constant factor, and λ represents the incident X-ray wavelength.

Figure 1c shows the crystal structure of KAlSiO_4_. There are many types of KAlSiO_4_ space groups. The space group corresponding to KAlSiO_4_ synthesised in this study is P212121(19), which is an orthorhombic structure (α = β = γ = 90°) with unit cell parameters a = 0.9057 nm, b = 1.5642 nm, c = 0.8582 nm, and V = 1.2158 nm^3^. It can be seen from the figure that K^+^ is filled between the [SiO_4_] and [AlO_4_] tetrahedrons. K^+^ has a 7-coordinated 4a site with higher symmetry and a 10-coordinated 4a site with lower symmetry in KAlSiO_4_ crystals. The crystal structure along the z-axis has high symmetry. This is conducive to the narrowband emission of Tb^3+^.

Figure 1d,e show the refined XRD images of KAlSiO_4_:1.5 mol% Tb^3+^ and KAlSiO_4_:1.5 mol% Tb^3+^, 1.5% Li^+^, respectively. It can be seen from the refined diagram that the XRD refined results of this series of samples are reliable. It was further confirmed that Tb^3+^ and Li^+^ doping had no significant effect on the crystal structure of KAlSiO_4_.

**Figure 2:**
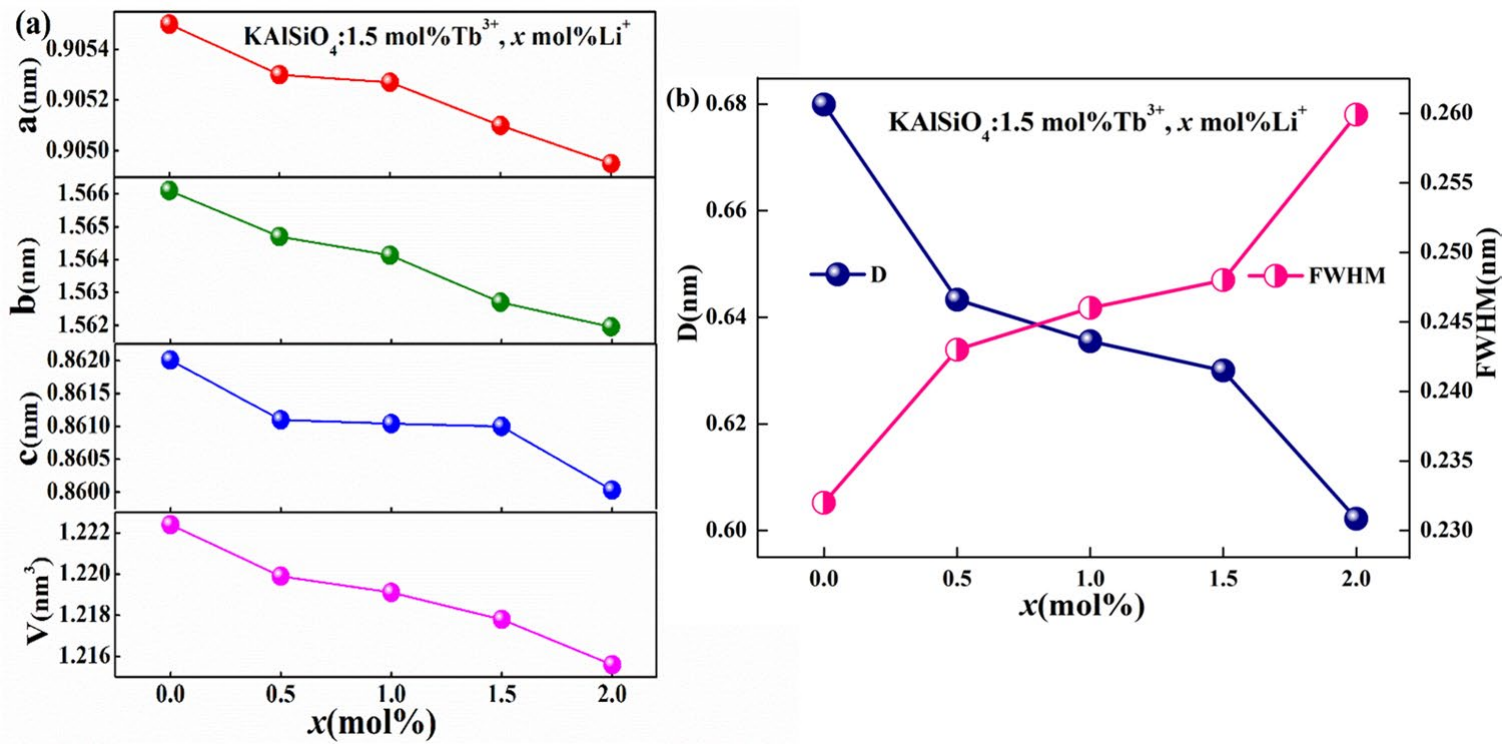
(**a**) Variation of the unit cell parameters with doping concentration (*x* = 0,0.5,1,1.5,2) for KAlSiO_4_: 1.5 mol% Tb^3+^, *x* mol% Li^+^ (**b**) The relationship between *x*, the grain size, and the (202) crystal plane FWHM in KAlSiO_4_: 1.5 mol% Tb^3+^, *x* mol% Li^+^**.**

Figure 2a shows the variation of the unit cell parameters of KAlSiO_4_: 1.5 mol% Tb^3+^, *x* mol% Li^+^ for various doping concentrations (*x* = 0, 0.5, 1, 1.5, 2). The changes in V, a, b, and c can be obtained from the refined XRD results. The relational formula [Eq. (2)]^9^ of the orthorhombic crystal plane spacing d (hkl) and crystal plane group (hkl) is used for the verification.

2 $$\frac{1}{{d^{2} }} = \frac{{h^{2} }}{{a^{2} }} + \frac{{k^{2} }}{{b^{2} }} + \frac{{l^{2} }}{{c^{2} }}$$

It can be seen from the figure that as the Li^+^ concentration increases, the values of V, a, b, and c decrease to varying degrees. When Tb^3+^ and Li^+^, which have a small radius, replaces the large radius of K^+^, the volume of the unit cell will shrink, causing the unit cell parameters to gradually show a downward trend.

Figure 2b shows the relationship between *x* in KAlSiO_4_: 1.5 mol% Tb^3+^, *x* mol% Li^+^ (*x* = 0, 0.5, 1, 1.5, 2), the grain size and the (202) full width at half maximum (FWHM) of the crystal plane. The grain size (D) is calculated using Eq. (3)^10, 11^.

3 $$D = \frac{K\lambda }{{W\cos \theta }}$$

Here, λ = 0.15406 nm and K = 0.89. W is the FWHM corresponding to the strongest diffraction peak, and θ is the angle of the strongest diffraction peak. With the increase in *x*, the grain size of the sample decreased by 11.4%, further confirming the shift of the XRD diffraction peak to the high-angle direction.

### Luminescence characteristics of KAlSiO_4_: ***x*** mol%Tb^3+^ series samples

**Figure 3:**
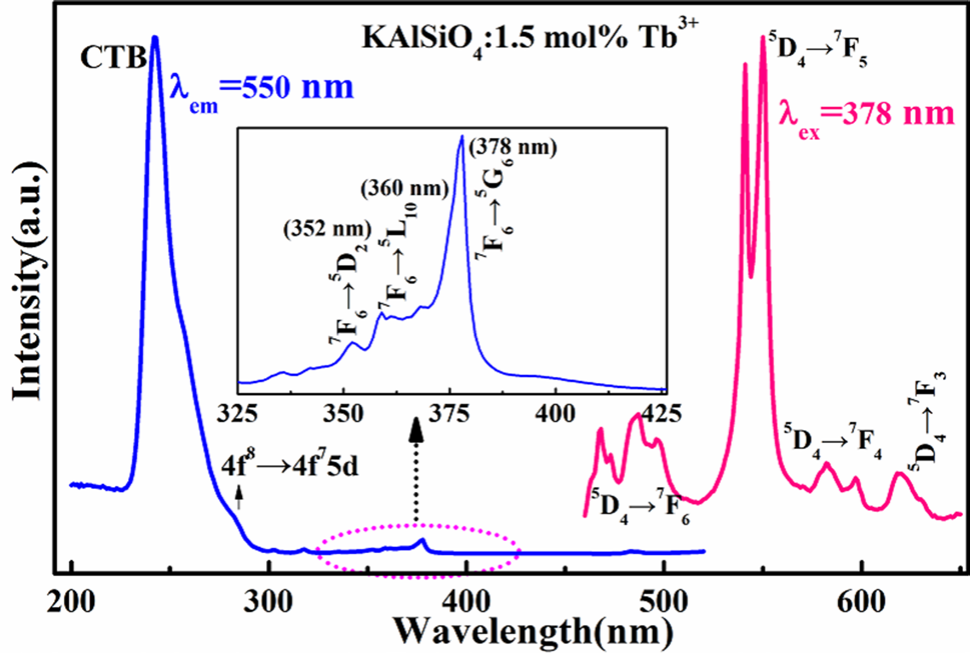
Excitation and emission spectra of KAlSiO_4_:1.5 mol% Tb^3+^ (the inset is an enlarged view of the excitation spectrum from 325–425 nm).

Figure 3 shows the excitation and emission spectra of the representative sample KAlSiO_4_: 1.5 mol% Tb^3+^. The inset is an enlarged view of the excitation spectrum in the range from 325 to 425 nm. Under the excitation of the characteristic excitation wavelength of Tb^3+^ at 378 nm, it can be seen from the figure that the strongest emission peak of the phosphor is at 550 nm, which belongs to the narrow-band green light emission. The phenomenon of spectral splitting occurs at 540 nm. This is because the 7 coordination of Tb^3+^ is substituted. K^+^ has a low symmetry position (Fig. 1c), which is affected by the crystal field environment. Compared with^12^, the strongest emission peak has a blue shift of 5 nm. Under 550 nm monitoring, a charge transfer band appeared in the range from 200 to 300 nm. There are different excitation bands from 200–500 nm, indicating that the sample can be effectively excited by ultraviolet light. The charge transfer band (CTB) of Tb^3+^ ions at 249 nm is attributed to the 4f.-5d transition of Tb^3+^ ion^7, 13^. Owing to the strong symmetry of potassium aluminosilicate belonging to the frame silicate series, the incorporated Tb^3+^ ions are less affected by the crystal field environment, and there is no obvious spectral split at 249 nm and 378 nm. When the sample was excited at 378 nm, it was found that the electrons in the ^7^F_6_ ground state of Tb^3+^ in the sample were excited to the ^5^D_J_ (J = 2, 3, 4) excited state. These electrons then relaxed to the ^5^D_4_ energy level by non-radiative transition, while some of the electrons undergo ^5^D_4_ to ^7^F_J_ (J = 6, 5, 4, 3) radiative transition^14^.

**Figure 4:**
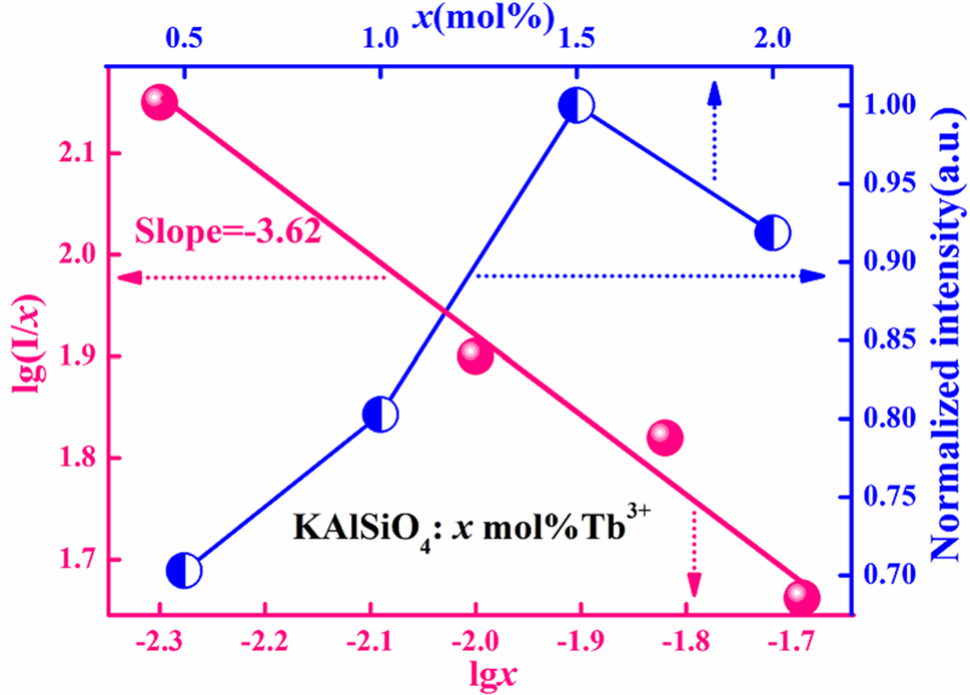
Concentration quenching mechanism diagram for KAlSiO_4_: *x* mol% Tb^3+^ (*x* = 0.5, 1, 1.5, 2).

Figure 4 is a diagram of the concentration quenching mechanism of KAlSiO_4_:*x* mol% Tb^3+^(*x* = 0.5, 1, 1.5, 2). It can be seen that when *x* = 1.5, the absorption intensity at 378 nm in the excitation spectrum is the strongest, resulting in the strongest emission intensity. The luminous intensity of the sample reaches its maximum, and concentration quenching occurs. When the concentration was increased beyond 1.5 mol%, the absorption intensity of each peak began to decrease slightly. The critical distance is calculated using Eq. (4)^15^:

4 $$R_{c} = 2\left( {\frac{3V}{{4\pi X_{e} N}}} \right)^{\frac{1}{3}}$$

R_c_ is the critical distance of concentration quenching, X_e_ is the critical concentration of Tb^3+^, V is the unit cell volume, and N is the number of unit cells. In this series of samples, V = 1.2158 nm^3^, X_e_ = 0.015, and N = 12. It is concluded that R_c_ = 2.35 nm, which is greater than 0.5 nm. (‘0.5 nm’ is an index that distinguishes exchange interaction and electrical multi-level interaction. When R_c_ > 0.5 nm, the concentration quenching mechanism is due to a multi-level interaction.) When a single activator is incorporated into the matrix, the relationship between the luminous intensity of the sample and the concentration of the activator *x* is as follows:

5 $$\lg ({I \mathord{\left/ {\vphantom {I x}} \right. \kern-\nulldelimiterspace} x}) = c - ({\theta \mathord{\left/ {\vphantom {\theta 3}} \right. \kern-\nulldelimiterspace} 3})lgx$$

*θ* is a multilevel interaction function, and c is a constant. n = 6, 8, and 10 represent dipole–dipole, dipole–quadrupole, and quadrupole–quadrupole interactions, respectively^16^. The sample is excited by near-ultraviolet light at 378 nm with *x* = 0.005, 0.01, 0.015, 0.02. The relationship between lg(*I/x)* and lg(*x*) at 550 nm is shown in Fig. 4. It was found that the fitting slope is -*θ*/3≈-3.62. Hence, *θ*≈10, which corresponds to the electric four-level interaction.

### Luminescence characteristics of KAlSiO_4_: 1.5 mol%Tb^3+^, ***x*** mol% Li^+^ series samples

**Figure 5:**
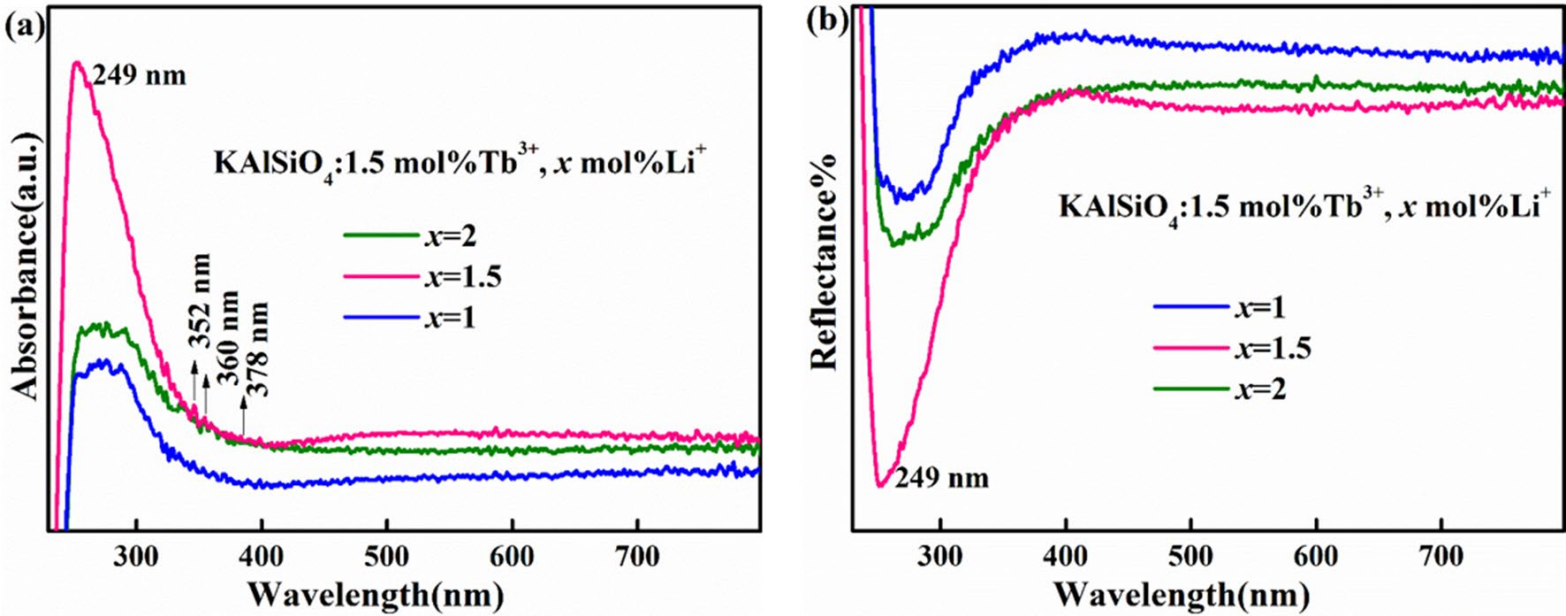
(**a**) UV–Vis absorption spectrum and (**b**) UV–Vis-NIR diffuse reflectance spectrum of KAlSiO_4_: 1.5 mol% Tb^3+^, *x* mol% Li^+^ (*x* = 1,1.5,2) series samples.

Figure 5a shows the UV–Vis absorption spectrum of the KAlSiO_4_: 1.5 mol% Tb^3+^, *x* mol% Li^+^ (*x* = 1, 1.5, 2) series samples. It can be seen from the figure that this series of samples absorbs UV light well in the 250–400 nm range. The strongest absorption peak at 249 nm is attributed to the charge transfer band transition (CTB) of Tb^3+^. The absorption peak at 283 nm is due to the 4f^8^ → 4f^7^5d^1^ transition^17^. Weak absorption peaks appear at 352, 360, and 378 nm, respectively. The characteristics of the absorption spectrum are consistent with those of the excitation spectrum of the KAlSiO_4_: 1.5 mol% Tb^3+^ sample. When *x* = 1, 1.5, and 2, three absorption peaks are obtained in the wavelength range from 300–400 nm, all of which are typical 4f.-4f. electronic transitions of Tb^3+^. When *x* = 1.5, the tangent of the absorption peak has the steepest slope. It shows that the KAlSiO_4_:1.5 mol% Tb^3+^, 1.5 mol% Li^+^ sample shows the strongest absorption of ultraviolet light in the wavelength range from 250–800 nm. The excitation light of a commercial ultraviolet LED chip can effectively excite the sample. The strong absorption at 378 nm corresponds to the^7^F_6_ → ^5^G_6_ transition, and the other two weak absorptions at 352 nm and 360 nm originate from the^7^F_6_ → ^5^D_2_ and^7^F_6_ → ^5^L_10_ transitions, respectively. The results of UV–visible absorption spectroscopy showed that Tb^3+^ activator ions and Li^+^ were successfully incorporated into KAlSiO_4_. This result is consistent with the XRD results.

Figure 5b shows the UV–Vis-NIR diffuse reflectance spectrum of the KAlSiO_4_:1.5 mol% Tb^3+^, *x* mol% Li^+^ (*x* = 1, 1.5, 2) series samples. It can be seen from the figure that the sample has strong absorption in the wavelength range from 250–400 nm, and the sample with *x* = 1.5 has the strongest absorption of ultraviolet and near-ultraviolet light. This result is consistent with the results of Figs. 3 and 5a. This further proves that this series of samples is suitable for excitation by white light LED chips. The strongest absorption peak at 249 nm is attributed to the 4f-5d charge transfer band transition of Tb^3+^ ion^14^. The absorption of the 4f-4f transition is weaker than that of the CTB transition.

**Figure 6:**
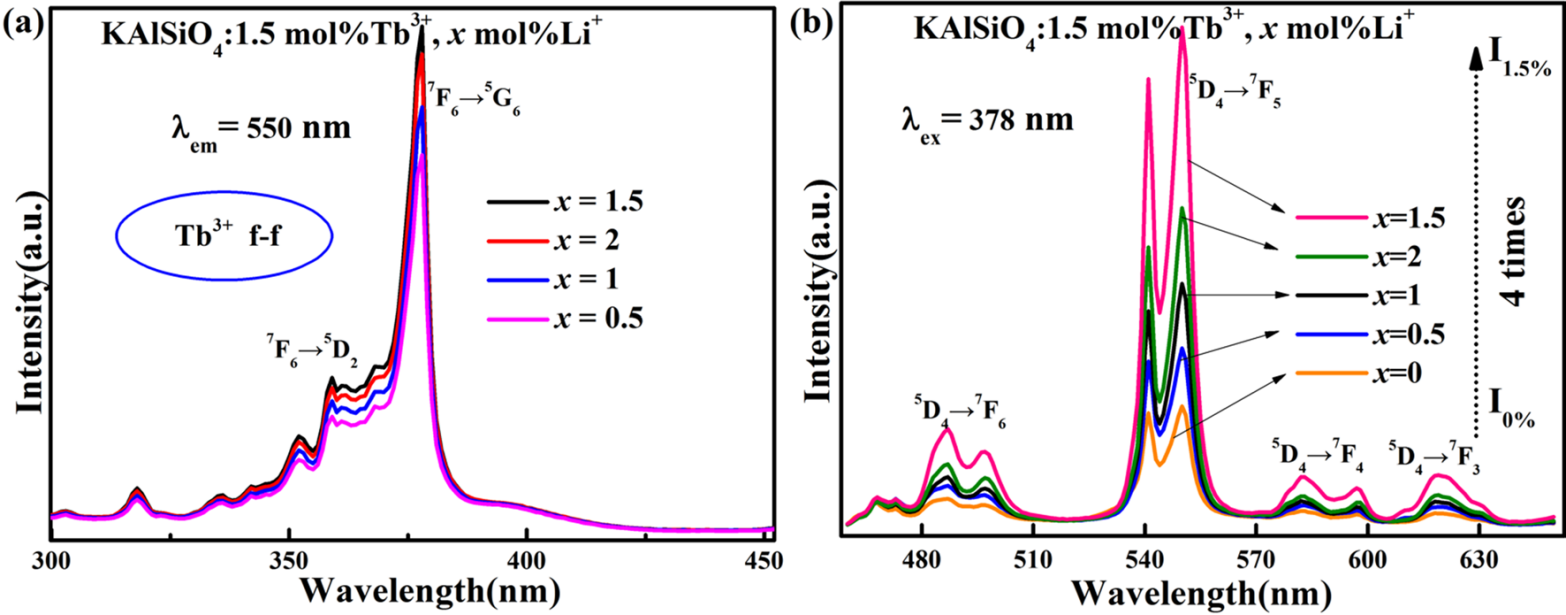
(**a**) Excitation spectrum and (**b**) emission spectrum of KAlSiO_4_: 1.5 mol% Tb^3+^, *x* mol% Li^+^ series samples.

Figure 6a,b shows the excitation spectrum and emission spectrum of the KAlSiO_4_:1.5 mol% Tb^3+^, *x* mol% Li^+^ series samples. The excitation spectrum of this series of samples is obtained by monitoring with Tb^3+^ at 550 nm (^5^D_4_ → ^7^F_5_). There are a series of excitation bands in the range of 300-450 nm, the main peaks are respectively 318, 352, 359, 378 nm, of which the strongest peak is at 378 nm. It confirms that it can be excited by ultraviolet light, near ultraviolet light and blue light.

It can be seen that as *x* increases, the intensity of the strongest emission peak at 550 nm (when excited with 378 nm) initially increases and then decreases. The best luminescence intensity is achieved when *x* = 1.5 and is approximately 4 times the luminous intensity of the KAlSiO_4_:1.5 mol% Tb^3+^ sample, which can further prove that after Li^+^ is added to the lattice, it promotes lattice matching. When *x* = 1.5, the quenching concentration of KAlSiO_4_:1.5 mol% Tb^3+^, *x* mol% Li^+^ can be obtained. At the same time, Li_2_CO_3_ can be used both as a charge compensation agent and as a flux. When Li_2_CO_3_ is used as a flux, Li^+^ can modulate the valence of Tb^3+^ ions, and it can contributes to the good light-emitting performance of the phosphor, that is to say, when the doping concentration of Tb^3+^ increases to 2 mol%, the luminescence performance of the sample is declined.

The substitution of K^+^ with Tb^3+^ is an unequal valency substitution. Doping with a small amount of Li^+^ can improve the lattice matching efficiency as it belongs to the same family as K^+^. This makes the cation charge in the crystal reach a relatively balanced state, thereby promoting the successful entry of Tb^3+^ into the K^+^ lattice site, and greatly enhancing the luminous efficiency of the strongest emission peak. This result shows that the incorporation of Li^+^ is beneficial for improving the luminous performance of the new KAlSiO_4_:1.5 mol% Tb^3+^ green-emitting phosphor. Hence, the KAlSiO_4_:1.5 mol% Tb^3+^, 1.5 mol% Li^+^ green-emitting phosphor is expected have higher luminous efficiency.

**Figure. 7:**
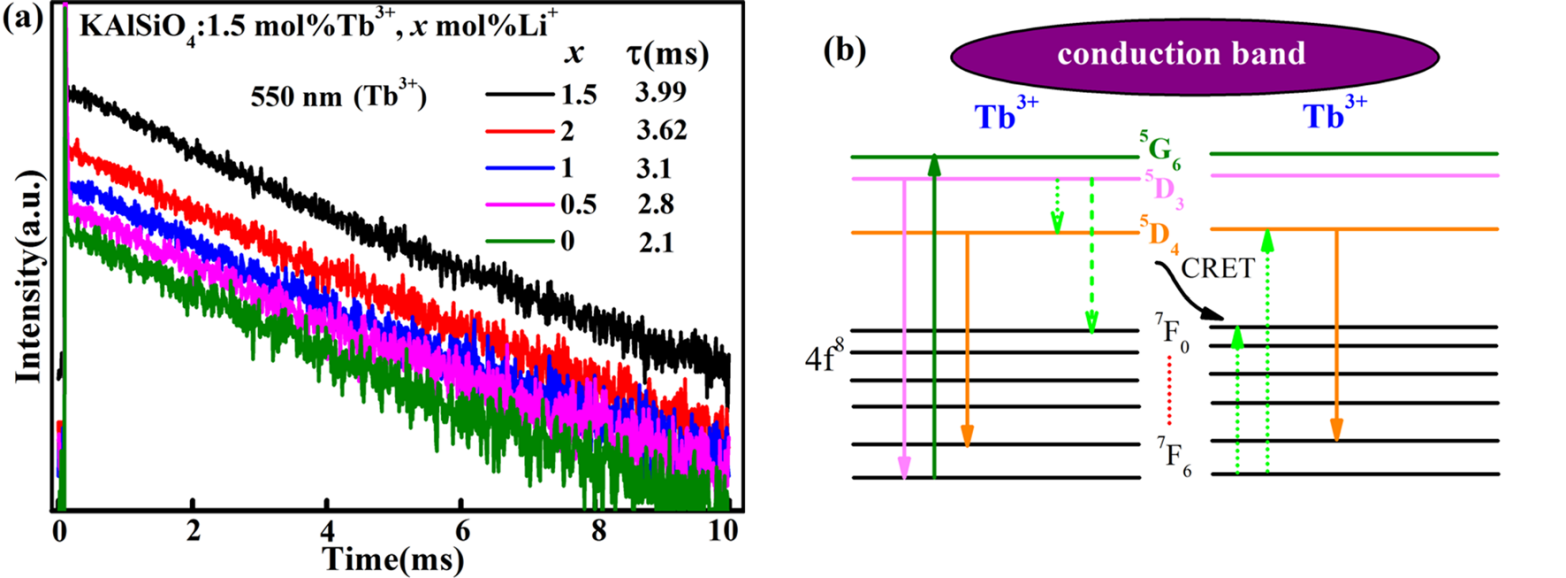
(**a**) Lifetime decay curves of KAlSiO_4_:1.5 mol% Tb^3+^, *x* mol% Li^+^ series samples at 550 nm; (**b**) the energy level diagram of Tb^3+^ in KAlSiO_4_.

Figure 7a shows the lifetime decay curves of the KAlSiO_4_:1.5 mol% Tb^3+^, *x* mol% Li^+^ series samples at 550 nm. It can be seen that as the Li^+^ concentration increases, the Tb^3+^ lifetime gradually increases. When *x* = 1.5, the lifetime value reaches the maximum, which is 3.99 ms. This lifetime value is 2.45 ms longer than that reported in^18^. The lifetime decay curve is completely consistent with the emission spectrum shown in Fig. 6, indicating that for the KAlSiO_4_ matrix, the best doping concentration of both Tb^3+^ and Li^+^ is 1.5 mol%, which results in the highest luminous efficiency and the longest lifetime. It is suitable for use in white LED lighting. The lifetime value is calculated using Eq. (6)^19^:

6 $$\tau^{ * } = \frac{{A_{1} \tau_{1}^{2} + A_{2} \tau_{2}^{2} }}{{A_{1} \tau_{1} + A_{2} \tau_{2} }}$$

Figure 7b shows the energy level diagram of Tb^3+^ in KAlSiO_4_. The energy transfer between Tb^3+^-Tb^3+^ generally occurs in this way. For example, in this system, the excited state electrons will quickly relax to the lower 4f. state, such as^5^D_3_,^5^D_4_, and then return to ground state.

**Figure 8:**
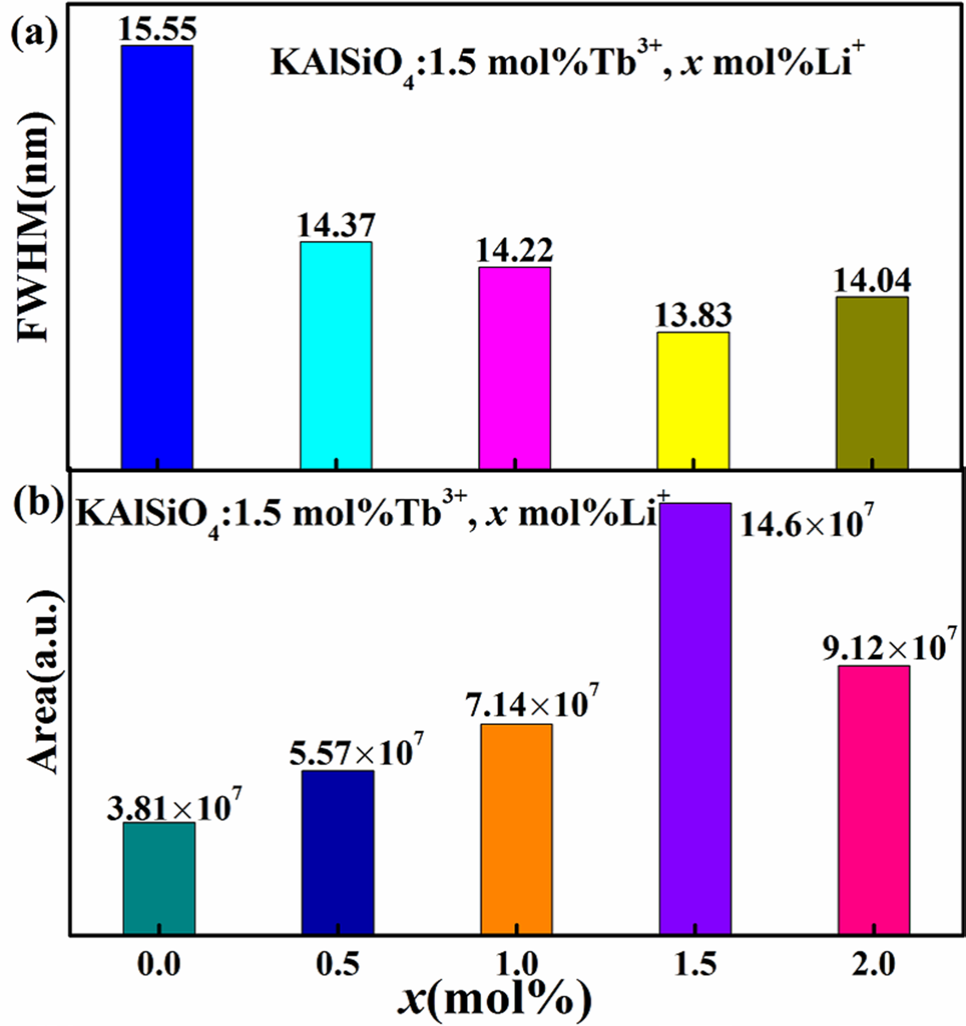
(**a**) Relationship between *x* and FWHM at 550 nm for KAlSiO_4_: 1.5 mol% Tb^3+^, *x* mol% Li^+^ sample; (**b**) relationship between *x* and the integrated peak area at 550 nm for KAlSiO_4_: 1.5 mol% Tb^3+^, *x* mol% Li^+^ sample.

Figure 8a, b shows the relationship between *x* and the FWHM and the integrated peak area at 550 nm for the KAlSiO_4_:1.5 mol% Tb^3+^, *x* mol% Li^+^ series samples. It can be seen from the figure that when *x* = 1.5, the FWHM value is minimised, which is suitable for backlight LED lighting. The integrated peak area intensity reaches a maximum, which is consistent with the emission spectrum characteristics shown in Fig. 6.

**Figure 9:**
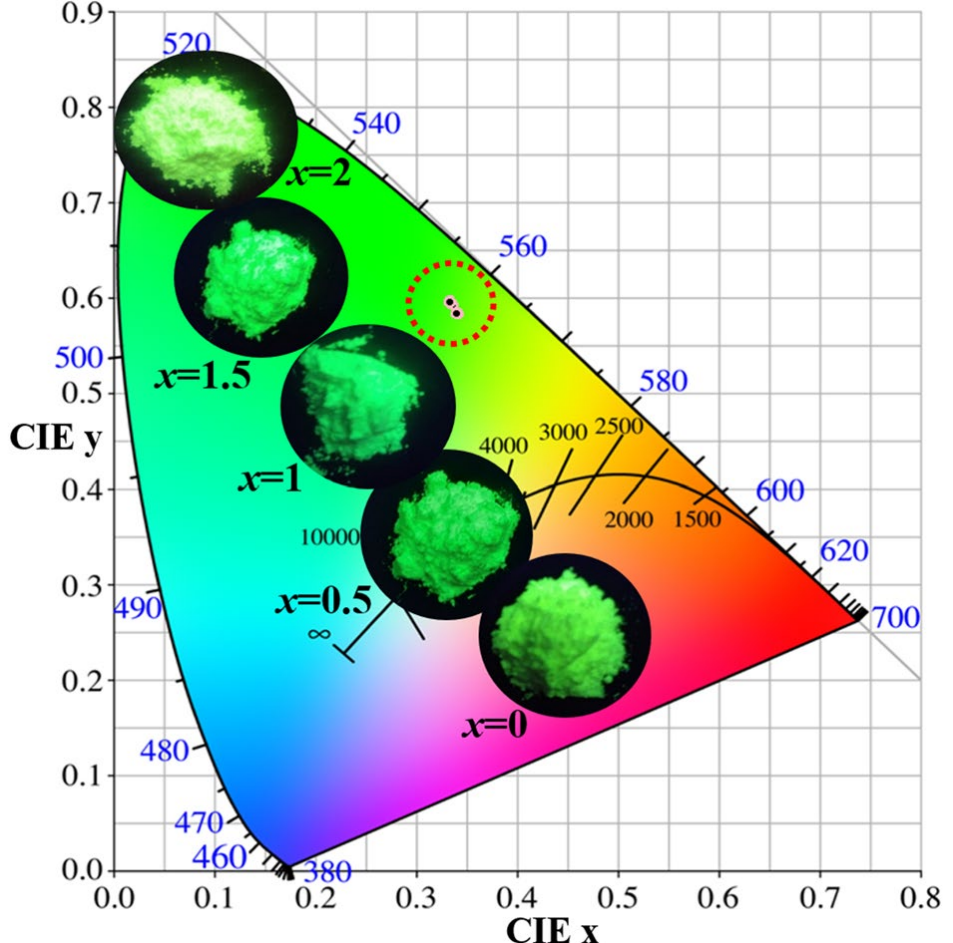
Colour coordinates and body colour changes of the KAlSiO_4_: 1.5 mol% Tb^3+^, *x* mol% Li^+^ series samples.

Figure 9 shows the colour coordinates and body colour changes of the KAlSiO_4_:1.5 mol% Tb^3+^, *x* mol% Li^+^ series samples. It can be seen that the colour coordinates of the samples do not change significantly with the increase in *x*. The colour coordinates of all the samples in the series are near the green region (0.33, 0.59), which preliminarily shows that the series of samples has good colour stability. The body colour of the sample gradually changes to deep green as *x* increases.

Table 1 shows the colour coordinates, colour purity, internal quantum yield (IQY), and colour temperature (CCT) of the KAlSiO_4_:1.5 mol% Tb^3+^, *x* mol% Li^+^ series samples. It can be seen that the coordinates of all the samples are approximately (0.32, 0.56). The internal quantum yield is as high as 42%, the colour purity is as high as 83.3%, and the colour temperature of the samples is approximately 5500 K. The colour purity is calculated using Eq. (7)^20^:

7 $$C_{p} = \frac{{\sqrt {(x_{s} - x_{i} )^{2} + (y_{s} - y_{i} )^{2} } }}{{\sqrt {(x_{d} - x_{i} )^{2} + (y_{d} - y_{i} )^{2} } }} \times 100\%$$

*C*_*P*_ is colour purity, (*x*_*d*_, *y*_*d*_) = (0.3473, 0.65) is the international standard colour coordinate corresponding to the main wavelength of 550 nm, (*x*_*i*_, *y*_*i*_) = (0.333, 0.333) is the colour coordinate of standard white light, and (*x*_*s*_, *y*_*s*_) is the colour coordinate of the series of samples. It can be seen from Table 1 that when *x* = 1.5, the sample colour purity is 83.3% and the quantum yield is 42%.

**Table 1:** Colour coordinates, colour purity, colour temperature, and internal quantum yield of KAlSiO_4_: 1.5 mol % Tb^3+^, *x* mol % Li^+^ samples.

*x*	CIE(x, y)	C_P_	CCT	IQY
0	(0.3399, 0.5838)	79.06%	5461	18%
0.5	(0.3417, 0.5839)	79.15%	5430	23%
1	(0.3359, 0.5927)	81.85%	5533	27%
1.5	(0.3327, 0.5974)	83.3%	5589	42%
2	(0.3331, 0.596)	82.8%	5582	35%

**Figure 10:**
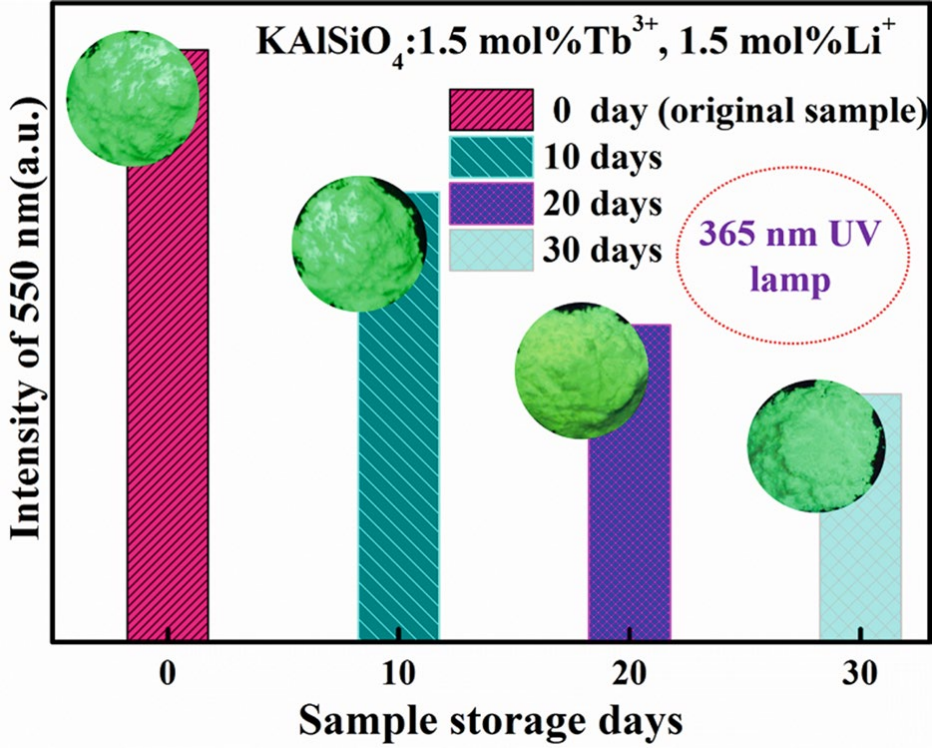
Variation of the emission peak intensity and sample body colour of KAlSiO_4_:1.5 mol% Tb^3+^, 1.5 mol% Li^+^ with number of storage days.

Figure 10 shows the emission spectrum of the KAlSiO_4_: 1.5 mol% Tb^3+^, 1.5 mol% Li^+^ when it is stored and exposed to air for 0, 10, 20, and 30 days. The relationship between the sample body colour and the number of storage days is also shown in the figure. It can be seen from the figure that the emission intensity is gradually weakened. However, the luminous intensity is reduced by half, and the sample main body colour still emits green light when irradiated with a 365 nm UV lamp. This further shows that the new green-emitting fluorescent phosphor has good stability and is suitable for the green component of the three-primary white LED.

**Figure 11:**
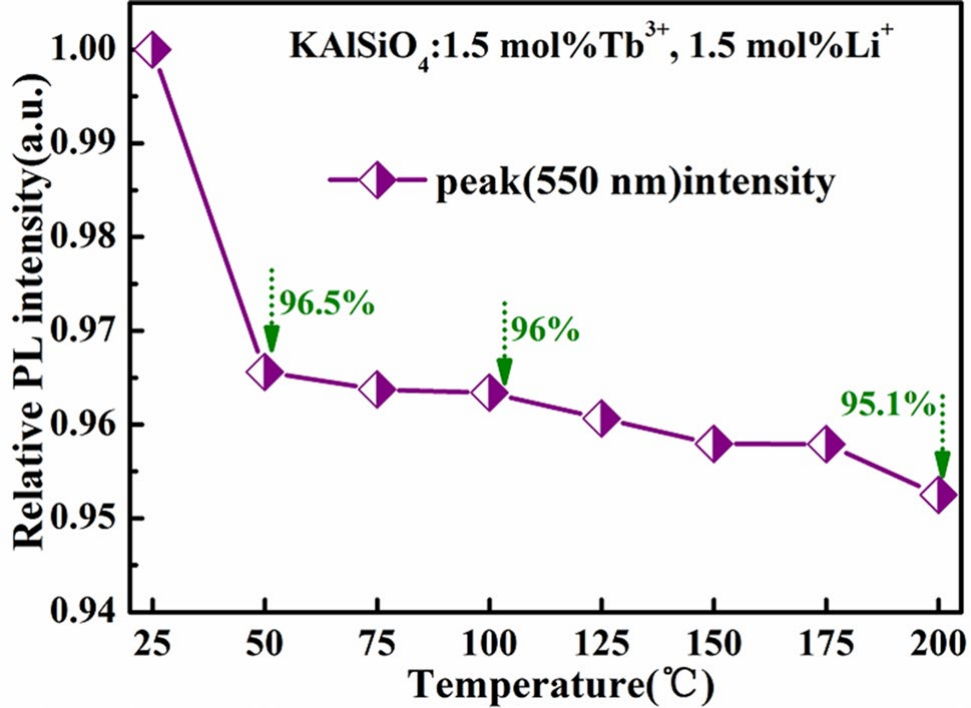
Variation of spectral light intensity of KAlSiO_4_:1.5 mol% Tb^3+^, 1.5 mol% Li^+^ with temperature.

The luminous performance of the phosphor suitable for white LED devices is generally determined by measuring the thermal stability of the phosphor. Figure 11 shows the variation of the intensity of the spectrum corresponding to KAlSiO_4_: 1.5 mol% Tb^3+^, 1.5 mol% Li^+^ with temperature. It can be seen from the figure that when the temperature rises, the emission peak intensities of the phosphor at 550 nm at 50 °C, 100 °C, and 200 °C are 96.5%, 96%, and 95.1% at room temperature, respectively. This shows that the silicate phosphor has good luminous efficiency and good thermal stability. This shows that the luminescence performance of the silicate phosphor has room for improvement. It is expected to be suitable for green phosphors in LED devices for a new generation of solid-state lighting.

## Conclusion

We have prepared a new green-emitting phosphor, KAlSiO_4_:1.5 mol %Tb^3+^, *x* mol% Li^+^ (*x* = 0, 0.5, 1, 1.5, 2). The chemical structure of this series of samples is found to be highly stable. The doping of Tb^3+^ and Li^+^ does not affect the crystal structure of the matrix. After the small-radius rare earth ions replace K^+^, the grain size decreases by 11.4% with an increase in the concentration of Li^+^ doping. The optimal doping concentration of Li^+^ in KAlSiO_4_:1.5 mol% Tb^3+^ was found to be 1.5 mol%. When *x* = 1.5, the sample strongly absorbs the ultraviolet light in the range from 250 to 400 nm. The luminous intensity of the KAlSiO_4_:1.5 mol% Tb^3+^ sample at 550 nm is increased by 4 times. The sample lifetime increased to 3.99 ms, the integrated peak area at 550 nm reached the maximum, and the full width at half maximum dropped to 13.83 nm. These results are closely related to the highly symmetrical crystal structure of the sample matrix. The colour purity of the sample series was found to be 83.3%. The body colour of the samples showed a little change with the increase in storage days. The samples exhibited good colour stability and thermal stability. The experimental results show that the developed phosphor is expected to become a candidate material for the green component of solid-state lighting white LEDs.

## Experiment

### Sample preparation and characterisation

KAlSiO_4_:1.5 mol% Tb^3+^, *x* mol% Li^+^ (*x* = 0.5, 1, 1.5, 2) was prepared in an MF-1750C Beyke high-temperature box furnace by the high-temperature solid-phase method at 1350 °C for 210 min. The main raw materials used are listed in Table 2. The Li_2_CO_3_ can act as a flux, which contributes to the favorable properties of the KAlSiO_4_:1.5 mol% Tb^3+^ phosphors in this work^1^.

**Table 2:** Raw materials of KAlSiO_4_: Tb^3+^, Li^+^ phosphor.

Chemical formula of raw materials	Purity	Production company
K_2_CO_3_	99.99%	Shanghai Aladdin Biochemical Technology Co., Ltd
Al_2_O_3_	99.99%	
SiO_2_	99.99%	
Tb_2_O_3_	99.9%	
Li_2_CO_3_	99.99%	

The quality of all the raw materials was calculated by the stoichiometric molar ratio method (mol%). The weighed materials were placed into an agate mortar and ground for 35 min. The uniformly mixed series of samples were placed into corundum crucibles and calcined in a high-temperature box furnace at a heating rate of 5 °C/min. After cooling the sample to room temperature, it was removed and ground again for the sample loading test.

The instrument parameters and experimental methods used in this study are presented in Table 3.

**Table 3:** Instrument parameters and experimental methods used in this study.

Experimental method	Test instrument name (or software name)
Phase analysis	Shimadzu XRD-6100 powder diffractometer
Photoluminescence spectrum (lifetime)	FLS920 Steady/Transient Fluorescence Spectrometer, Edinburgh, UK
Color coordinates	CIE1931
Internal quantum yield(IQY)	Hamamatsu C11347 absolute quantum efficiency tester
Thermal stability	Beijing (FJ-427A1) tester
